# Glucose Metabolism Modulation as a Strategy to Enhance Cancer Radiotherapy

**DOI:** 10.3390/metabo15120793

**Published:** 2025-12-12

**Authors:** Shuaining Gao, Xiaochang Liu, Shi Chen, Pingkun Zhou

**Affiliations:** 1 School of Basic Medical Sciences, Hengyang Medical College, University of South China, Hengyang 421001, China; gsn0991@163.com; 2 Department of Radiation Biology, Beijing Key Laboratory for Radiobiology, Beijing Institute of Radiation Medicine, Beijing 100850, Chinachace1215@163.com (S.C.)

**Keywords:** glucose metabolic reprogramming, warburg effect, glycolysis, radioresistance, radiosensitization targets

## Abstract

A systematic literature review of the PubMed database, filtering for publication dates up to and including October 2025, was conducted to identify relevant studies on glucose metabolism and radiotherapy. Radioresistance poses a major therapeutic challenge, in which tumor-associated glucose metabolic reprogramming, characterized by the Warburg effect, supports cellular energy requirements and contributes to radioresistance by facilitating DNA repair and promoting survival pathways. Targeting pivotal glycolytic enzymes, such as hexokinase (HK) and pyruvate kinase M2 (PKM2), and integrating radiotherapy with metabolic modulators have been shown to improve radiosensitivity. Special emphasis is placed on how these interventions remodel the tumor microenvironment and modulate antitumor immunity—emerging factors that influence therapeutic efficacy. This review highlights mechanistic insights and potential therapeutic targets for the development of effective radiosensitization strategies.

## 1. Introduction

Cancer cells fundamentally rewire their metabolic pathways to sustain proliferation and survival, with altered metabolism now recognized as a core hallmark of malignancy. This metabolic reprogramming critically influences therapeutic responses, including resistance to radiotherapy [1]. Glucose metabolic reprogramming, characterized by increased glycolysis and lactate production under normoxic conditions (Warburg effect), supports tumor adaptation to stress [2,3]. It increases radioresistance by promoting DNA repair and survival pathways. This process affects the response to radiation by altering redox balance and energy metabolism [4,5,6]. Some tumors escape glucose metabolism-mediated radioresistance regulation by activating fatty acid synthesis [7]. Metabolites such as lactate and glutamine also contribute to immune evasion [8,9,10].

In glioblastoma, radiotherapy triggers a metabolic shift toward fatty acid β-oxidation, contributing to radioresistance and promoting immune evasion by regulating CD47 [11]. Inhibiting glycolytic transporters (such as GLUT1/4) or metabolic enzymes (including LDHA, HK2, and PKM2) has been shown to significantly improve radiosensitivity [12,13,14,15,16,17,18,19].

Consequently, targeting glucose metabolic reprogramming has become a crucial strategy for enhancing tumor radiosensitivity. This review systematically elucidates the key mechanistic roles of glucose metabolic reprogramming in tumor radioresistance, focusing on its role in DNA damage repair, cell cycle regulation, and tumor microenvironment remodeling. Furthermore, we critically evaluate potential therapeutic strategies targeting key glycolytic pathways to improve radiosensitivity, providing novel insights for developing more effective radiotherapeutic interventions.

## 2. Overview of Reprogramming of Glucose Metabolism

### 2.1. Basic Concepts and Biological Significance of Glucose Metabolic Reprogramming

Glucose metabolic reprogramming, a hallmark of cancer, refers to alterations in metabolic pathways that enable cancer cells to meet heightened demands for energy, biosynthetic precursors, and redox balance to support rapid proliferation and survival [20,21]. A hallmark of this reprogramming is the upregulation of glycolysis coupled with a reduction in mitochondrial oxidative phosphorylation (OXPHOS) [22,23]. This shift plays a critical role in the pathophysiology of multiple disorders, especially cancer and neurodegenerative diseases.

Under physiological conditions, cells predominantly produce ATP through mitochondrial OXPHOS. In contrast, during glycolytic reprogramming, ATP generation shifts toward glycolysis despite adequate oxygen availability. This phenomenon, termed the Warburg effect, is often observed in tumor cells [24]. For example, during the induction of induced pluripotent stem cells (iPSCs), elevated glycolytic activity supports somatic cell reprogramming, while its suppression impairs reprogramming efficiency [25]. Similarly, in ischemic stroke, activated microglia undergo a metabolic transition from OXPHOS to glycolysis, a shift that significantly influences their functional behavior [26].

Reduced mitochondrial OXPHOS is a critical aspect of glycolytic reprogramming. Evidence indicates that OXPHOS activity is significantly diminished in tumor cells and in the context of neurodegenerative diseases [27]. For instance, during ischemic stroke, microglial metabolic reprogramming impairs mitochondrial function, leading to decreased ATP generation and elevated oxidative stress, which collectively aggravate neuroinflammation and neuronal injury [28,29]. These findings provide mechanistic insights into glycolytic reprogramming across diverse pathological conditions and offer a conceptual framework for the development of targeted therapeutic interventions.

### 2.2. Glucose Metabolism and Tumor Relationship

Altered glucose metabolism facilitates tumor progression by upregulating glycolytic pathways, leading to increased glucose uptake and lactate production to support the energy requirements of rapid proliferation [30]. In certain tumor types, reprogramming of fructose metabolism plays a pivotal role. Tumor cells use GLUT5 to import fructose, which promotes survival, proliferation, and enhanced migratory potential [31]. Furthermore, fructose metabolism contributes to tumor growth and metastasis by stimulating angiogenesis [32].

Altered glucose metabolism is closely linked to tumor progression. A study from Lanzhou University showed that KRAS mutation-driven metabolic alterations, including elevated glycolysis, glutamine utilization, and lipid biosynthesis, promote cancer cell proliferation and survival [33]. Moreover, glucose metabolic reprogramming in tumor-associated macrophages (TAMs) influences tumor cell migration, invasion, and angiogenic activity [34]. These findings underscore the critical role of glucose metabolic reprogramming across all stages of tumor development and highlight its potential as a therapeutic target [35,36].

## 3. Glucose Metabolism and the Cellular Response to Radiation Damage

Glycolytic reprogramming plays a key role in modulating tumor cell responses to radiation-induced stress. Under hypoxic and nutrient-limited conditions, tumor cells enhance glycolysis and lactate production to maintain proliferation. This metabolic adaptation also promotes resistance to radiotherapy by modulating oxidative stress and facilitating DNA damage repair pathways [36,37]. Therefore, understanding the interplay between glucose metabolism and cellular responses to radiation is essential for advancing radiosensitization strategies.

Recent studies have highlighted the essential role of glycolytic reprogramming in modulating cellular responses to radiation. Investigators from the University of South China and Central South University reported that TAB182 regulates lactate dehydrogenase A (LDHA) transcription, modulating glycolysis and lactate production and thereby contributing to tumor radioresistance [38]. Exposure to low-glucose conditions combined with palmitic acid significantly suppresses colorectal cancer cell proliferation and increases radiosensitivity by promoting reactive oxygen species (ROS) generation and inducing DNA damage.

### 3.1. Glucose Metabolism and DNA Damage Repair

#### 3.1.1. High Glycolysis Promotes DNA Damage Repair

DNA damage repair involves a series of enzymatic processes that restore damaged DNA, preserving genomic integrity and stability. The primary repair mechanisms include base excision repair, nucleotide excision repair, mismatch repair, homologous recombination (HR), and non-homologous end joining (NHEJ) [39,40,41,42]. These pathways are fundamental to the cellular response to environmental stressors such as radiation and chemical exposure and are vital for maintaining normal physiological functions.

Emerging evidence indicates a strong association between glycolytic reprogramming (particularly hyperglycolysis) and DNA damage repair [43,44]. Increased glycolytic activity enables tumor cells to increase glucose uptake and lactate production, supporting the high energy and biosynthetic demands of rapid proliferation. This metabolic shift not only drives tumor growth but also supports DNA repair by supplying key intermediates such as nucleotides and NAD^+^. For instance, the glycolytic enzyme aldolase A (ALDOA) has been shown to translocate to the nucleus following ionizing radiation, where it co-localizes with γ-H2AX and directly participates in double-strand break (DSB) repair [45]. Moreover, He Yulong’s group at Sun Yat-sen University demonstrated that lactate-mediated modification of the K388 residue on the DNA repair protein NBS1 enhances its recruitment to DSB sites, therefore promoting HR efficiency [46].

#### 3.1.2. Glucose Metabolic Pathways Regulate the DNA Damage Response

Alterations in glycolytic pathways play a critical role in regulating DNA damage responses, with metabolic enzymes and intermediates directly influencing the activity of DNA repair mechanisms. For example, proteasome activator subunit 3 (PSME3) promotes DNA repair in cervical cancer cells by upregulating glycolytic proteins, whereas PSME3 knockdown impairs glycolysis and elevates radiosensitivity [47]. Similarly, inhibition of integrin β5/focal adhesion kinase signaling following cisplatin treatment reduces glycolytic activity in breast and cervical cancer cells by downregulating glycolytic enzymes [48]. These observations suggest that metabolic reprogramming not only affects energy metabolism but also modulates radiosensitivity by regulating DNA damage repair pathways.

Poly(ADP-ribose) polymerase 1 (PARP1), a key mediator of DNA damage repair, relies on NAD^+^ as a substrate, the biosynthesis of which is dependent on glycolytic flux [49,50]. Glutamine metabolism also plays a pivotal role in supporting DNA repair processes [51,52]. Fu et al. [53] reported that radiation-resistant tumor cells show decreased glycolytic activity, mitochondrial respiration, and tricarboxylic acid (TCA) cycle flux, accompanied by increased glutamine anabolism. The silencing of glutamine synthetase impairs DNA repair kinetics and disrupts nucleotide biosynthesis, sensitizing tumor cells to radiotherapy.

### 3.2. Glucose Metabolism and Cell Cycle Regulation

The cell cycle [54,55,56,57] refers to the sequential progression of events from the completion of one cell division to the initiation of the next, a process that underlies cell growth, proliferation, and the preservation of tissue homeostasis. It is divided into four distinct phases: G1 (pre-DNA synthesis), S (DNA synthesis), G2 (post-DNA synthesis), and M (mitosis). Progression through these phases is tightly regulated by a network of molecular regulators, including cyclins, cyclin-dependent kinases (CDKs), and CDK inhibitors [58,59]. These factors function through specific complex formation to coordinate phase transitions, ensuring that cell division occurs only under favorable conditions.

Cell cycle progression requires significant energy input and biosynthetic resources. Glycolysis is a major energy-producing pathway, providing ATP through the catabolism of glucose by catalyzing it to pyruvate [60,61]. Throughout all phases of the cell cycle, glycolysis supplies energy and generates key intermediates such as glyceraldehyde-3-phosphate and phosphoenolpyruvate (PEP). These intermediates serve as precursors for the synthesis of nucleic acids, proteins, and lipids, supporting the biosynthetic demands associated with cell cycle progression [62,63,64,65].

Glycolytic metabolites influence cyclin activity and regulate cell cycle progression. For example, elevated lactate levels stabilize cyclin D1, facilitating the G1/S phase transition [66]. Hexokinase 2 (HK2) promotes cell cycle progression by enhancing glycolytic flux, which increases intracellular ATP and NAD^+^ levels, ultimately activating key cell cycle regulators [67].

Cell cycle regulators also exert control over glycolytic activity, establishing a bidirectional relationship between metabolism and cell proliferation. CDKs modulate the transcription of glycolytic genes, either by influencing transcription factors or through direct phosphorylation of gene promoters. CDK4/6–Cyclin D complexes activate E2F transcription factors, leading to the upregulation of glycolytic enzymes such as HK2 and LDHA, thereby increasing glycolytic flux [68]. Similarly, cell division cycle-associated protein 7 (CDCA7) promotes glycolysis, facilitating pancreatic cancer cell proliferation and chemoresistance [69]. This reciprocal regulation enables flexible metabolic adaptation to meet the energetic and biosynthetic requirements of cell cycle progression.

## 4. Glucose Metabolism and Tumor Radioresistance

### 4.1. Tumor Radioresistance and Intrinsic Factors

Radiotherapy remains a cornerstone of cancer treatment. It is utilized in >50% of cancer patients for its ability to achieve local tumor control while preserving organ function [70,71]. Its efficacy is limited by intrinsic or acquired tumor radioresistance, a critical obstacle to curative outcomes [72]. Tumor radioresistance refers to the ability of tumor cells to withstand radiation-induced cytotoxicity and often results in reduced radiotherapy efficacy or treatment failure [73,74,75]. This phenomenon arises from inherent cellular characteristics or adaptive responses triggered by radiation exposure. A major obstacle to successful radiotherapy, radioresistance, is the product of multifaceted mechanisms driven by both intrinsic and extrinsic factors within the TME.

**Figure 1:**
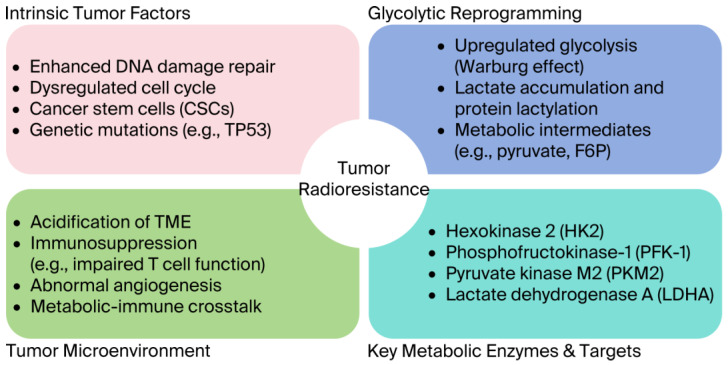
Factors affecting tumor radioresistance.

The intrinsic mechanisms of tumor radioresistance include a range of cellular adaptations that diminish the efficacy of radiotherapy (Figure 1). A central mechanism involves tumor cells’ increased capacity for DNA damage repair. Radiotherapy primarily exerts its cytotoxic effects via DNA DSBs, which some tumor cells can efficiently repair by activating key repair pathways, such as HR and NHEJ, increasing their survival rate after radiation exposure [76,77,78]. Dysregulation of the cell cycle also contributes significantly to intrinsic radioresistance. Aberrant activation of CDKs or altered cell cycle distribution may result in a predominance of tumor cells in radiation-insensitive phases during treatment, such as the G2 phase, reducing radiosensitivity [79,80,81]. Cancer stem cells (CSCs) are another intrinsic factor contributing to radioresistance. Characterized by self-renewal and differentiation capabilities, CSCs exhibit heightened resistance to radiation through the maintenance of stemness, the activation of specific signaling pathways, including the PI3K/AKT/mTOR axis, and metabolic reprogramming [82,83,84,85]. Metabolic adaptation is a further determinant of intrinsic radioresistance. Under radiotherapy-induced stress, tumor cells may rewire their metabolism, upregulating glycolysis or promoting fatty acid oxidation to support survival. For example, upregulation of fatty acid synthase (FASN) has been shown to stimulate fatty acid metabolism, thereby increasing resistance to radiation [86]. Genetic alterations also underlie radioresistant phenotypes. Mutations in tumor suppressor genes such as TP53 are frequently observed across tumor types and are closely linked to radioresistance. In head and neck squamous cell carcinoma and colorectal cancer, TP53 mutations are associated with reduced radiosensitivity, likely due to impairment of DNA damage response pathways [87,88]. Aberrant activation of YAP, a key effector of the Hippo signaling pathway, has been shown to increase DNA repair capacity and promote tumor cell proliferation, further contributing to radioresistance [89].

### 4.2. Role of Glycolysis in Radiotherapy Resistance

#### 4.2.1. Glycolytic Pathways Support Tumor Survival

The Warburg effect supports rapid tumor proliferation and influences signaling pathways through metabolite-mediated mechanisms that contribute to radioresistance [90,91,92]. Elevated glycolytic activity also mitigates radiotherapy-induced apoptosis by modulating redox balance and reducing intracellular ROS levels [93].

#### 4.2.2. Lactate Accumulation and Radiotherapy Resistance

Elevated glycolysis leads to the accumulation of metabolites such as lactate, which plays a regulatory role in DNA damage repair through protein lactylation, ultimately diminishing the efficacy of radiotherapy. In glioblastoma, ALDH1A3 activates PKM2, promoting lactate accumulation. Lactate-induced modification of XRCC1 increases DNA repair capacity, contributing to radioresistance [94]. Lactate dehydrogenase (LDH, which catalyzes the conversion of pyruvate to lactate) is frequently overexpressed in tumors and is associated with radioresistant phenotypes [38,95]. LDH facilitates radioresistance by increasing lactate production, thereby supporting tumor cell survival and proliferation. In therapy-resistant tumors, LDHA expression is significantly elevated and is accompanied by excessive lactate accumulation [96]. By catalyzing the conversion of pyruvate to lactate, LDHA accelerates glycolysis, providing both energy and biosynthetic intermediates necessary for sustaining rapid tumor growth and resisting radiotherapy.

#### 4.2.3. Other Metabolic Intermediates and Radiotherapy Resistance

Beyond increased glucose metabolism and lactate accumulation, glycolytic intermediates such as pyruvate, fructose-6-phosphate (F6P), and fructose-1,6-bisphosphate play critical roles in promoting radioresistance. These metabolites facilitate tumor cell survival and proliferation by regulating energy metabolism [97], increasing DNA repair mechanisms [98], modulating oxidative stress responses [99], and reshaping the immune microenvironment [100].

Pyruvate, a central glycolytic intermediate, plays a pivotal role in regulating tumor radioresistance by modulating the DNA damage response. Pyruvate improves FACT-mediated chromatin loading of γH2AX, promoting chromatin remodeling and facilitating efficient DNA repair. This mechanism contributes to increased glioblastoma resistance to radiation-induced DNA damage [101].

Pyruvate-metabolizing enzymes, including pyruvate kinase M2 (PKM2) and pyruvate dehydrogenase (PDH), are key regulators of metabolic reprogramming and radioresistance. PKM2 catalyzes the conversion of PEP to pyruvate with concurrent ATP generation during glycolysis. Beyond its enzymatic role, PKM2 modulates the accumulation of glycolytic intermediates, sustaining aerobic glycolysis in tumors that preferentially engage glycolysis despite sufficient oxygen availability [102,103,104,105]. Activation of PKM2 also induces the release of paclitaxel and indoleamine 2,3-dioxygenase (IDO) inhibitors, augmenting the efficacy of immunochemotherapy in non-small cell lung cancer (NSCLC) [106]. In comparison, PDH mediates the conversion of pyruvate to acetyl-CoA, facilitating its entry into the TCA cycle. Inhibition of PDH restores pyruvate carboxylase (PC) activity, leading to increased succinate secretion, which activates SUCNR1 signaling and boosts CD8^+^ T cell cytotoxic function, counteracting tumor-induced immunosuppression [107].

Phosphofructokinase-1 (PFK-1) catalyzes the conversion of F6P to fructose-1,6-bisphosphate, a key rate-limiting step in glycolysis that drives further ATP production [108]. Accumulation of F6P is associated with increased tumor radioresistance, reflecting its role in not only energy metabolism but also the regulation of multiple cellular processes. In hepatocellular carcinoma, radioresistant cells show elevated glucose uptake and increased F6P levels, promoting resistance to radiation through redistribution of glycolytic flux and increased branch-point anabolic activity [109].

### 4.3. Altered Tumor Microenvironment (TME) and Radiotherapy Resistance

#### 4.3.1. Acidification of the TME

Glycolytic reprogramming leads to excessive lactate production, resulting in acidification of the TME. The acidic TME significantly impairs radiotherapy efficacy by diminishing tumor cell radiosensitivity [110]. This effect is mediated through multiple mechanisms: the acidic TME directly subverts radiation-induced antitumor immunity by suppressing key chemokines such as CCL5, thereby blocking CD8^+^ T cell recruitment to tumors post-irradiation [111]. Research by Gu et al. revealed that an acidic TME activates the NF-κB pathway, inducing the release of inflammatory factors like IL-6, which promote tumor cell survival while further disrupting the immune microenvironment [112]. Within tumor cells, acidosis cooperates with hypoxia to maintain a state of constitutive ATM/CHK1/CHK2 DNA damage checkpoint activation, conferring markedly enhanced DNA repair capacity [113]. A nanotechnology system developed by Dou et al. showed that altering the TME—for instance, using nitric oxide release to alleviate hypoxia and acidosis—can synergistically regulate HIF1α and p53 expression, offering an effective strategy to reverse TME-mediated radioresistance [114]. Additionally, acidic conditions contribute to aberrant, permeable vasculature that further limits radiotherapy by impairing oxygen and drug delivery [115,116].

#### 4.3.2. Effects of Acidic Environment on Cellular Functions

This acidity adversely affects drug permeability and intratumoral distribution, resulting in subtherapeutic drug concentrations and diminished treatment efficacy. Furthermore, the stability of certain therapeutics is compromised under acidic conditions, further limiting their effectiveness. To address these challenges, pH-responsive drug delivery systems have emerged as a promising strategy. For instance, the CDBi, Mn-FA@CaP complex, a novel radiosensitizer, undergoes acid-triggered degradation, releasing smaller carbon dots capable of deep tumor penetration, thereby significantly increasing the efficacy of radiotherapy [117].

In addition to limiting drug permeability, acidic microenvironments contribute to tumor radioresistance through various mechanisms. Acidic conditions activate signaling pathways and increase DNA repair processes, reducing tumor cell radiosensitivity. In breast cancer, acidosis induces activation of β-catenin/TCF signaling, resulting in an increased proportion of CSCs with elevated stemness and migratory potential. This cellular phenotype facilitates tumor regeneration and recurrence after radiotherapy [118]. Moreover, acidic conditions suppress immune cell function and attenuate antitumor immune responses, further weakening radiotherapy-induced immune activation [119,120,121].

#### 4.3.3. Interaction Between Microenvironment and Metabolism

The interplay between the TME and metabolic reprogramming is pivotal in regulating tumor progression. Tumor cells adapt to hypoxic and nutrient-limited conditions by reprogramming their metabolism, particularly through upregulated glycolysis and glutamine metabolism, which support sustained proliferation and survival [122,123,124]. A central component of this adaptation involves the metabolic reprogramming of TAMs, which is closely linked to immunosuppression and tumor progression within the TME. Modulating TAM metabolism has been shown to augment antitumor immune responses and improve the efficacy of immunotherapy [125]. Tumor-derived metabolites, such as lactate and glutamine, further influence immune cell function, facilitating immune evasion. For example, lactate contributes to tumor progression by altering histone acetylation and suppressing macrophage inflammatory responses [126]. This metabolic–immune crosstalk orchestrates both tumor development and immune modulation within the TME.

Modulating the TME metabolism is a promising strategy to increase the efficacy of radiotherapy. A hallmark of the TME is its acidic pH, which is typically lower than that of normal tissues. pH-responsive nanomaterials have emerged as a potential tool for mitigating these limitations [127,128,129]. Targeting glutamine metabolism also disrupts the tumor energy supply and increases radiosensitivity by impairing tumor cell viability and simultaneously improving immune cell function through modulation of the immune microenvironment [52,130]. Further improvement in radiotherapy outcomes can be achieved by targeting key TME metabolites. Inhibiting LDHA, for example, reduces lactate accumulation and increases tumor cell sensitivity to radiation [131].

#### 4.3.4. Metabolic Crosstalk in the Tumor Microenvironment and Immune Synapse Function

Beyond fueling tumor growth, glucose metabolic reprogramming actively contributes to an immunosuppressive TME via metabolite-driven crosstalk. Impairment of T cell immune synapse function is a critical mechanism of radiotherapy resistance.

As a key glycolytic byproduct, lactate acts as a central mediator of immunosuppression. Elevated lactate concentrations acidify the TME, directly suppressing cytotoxic T lymphocyte (CTL) activity and proliferation. Lactate also promotes broad immunosuppression through epigenetic mechanisms such as histone lactylation, facilitating M2 macrophage polarization and impairing natural killer (NK) cell function [132]. This immunosuppressive environment critically attenuates the antitumor immune response initiated by radiotherapy.

Nutrient competition is another crucial mechanism. Activated T cells depend on glycolysis to support effector functions and clonal expansion. However, high glucose consumption by Warburg-effect-driven tumor cells results in local glucose deprivation. This metabolic stress directly compromises T cell immune synapse formation—an energy-intensive process that requires ATP for cytoskeletal dynamics and polarized granule secretion. Under glucose restriction, T cells display disrupted immune synapse integrity, diminished signaling transduction, impaired cytokine production (e.g., IFN-γ), and reduced tumor-killing activity [133].

#### 4.3.5. Key Glycolytic Enzymes as Hubs Linking Intrinsic Tumor Metabolism to the TME

The metabolic alterations within the TME are driven directly by the upregulated activity of specific glycolytic enzymes in tumor cells. For instance, LDHA overactivity is a primary driver of lactate accumulation, leading to TME acidification and promoting radioresistance through mechanisms such as protein lactylation [46,94]. Similarly, HK2-mediated high glycolytic flux not only fulfills the energy demands of cancer cells but also creates nutrient competition, impairing T cell function within the TME [133,134]. Furthermore, the PKM2 isoform, particularly in its low-activity state, favors metabolic reprogramming that sustains the Warburg effect and associated TME remodeling [102]. This direct link establishes these pivotal enzymes as high-priority therapeutic targets, whose inhibition is anticipated to simultaneously disrupt tumor cell metabolism and reverse the radioresistant TME, providing a foundation for the targeted strategies discussed in the following chapter.

## 5. Glucose Metabolic Reprogramming and Radiosensitization Targets

Building on the rationale that key glycolytic enzymes, such as HK2, LDHA, and PKM2, are central drivers of both tumor metabolism and the radioresistant TME, the following section focuses on therapeutic strategies directly targeting these molecules. Given the significant therapeutic obstacles posed by tumor complexity and heterogeneity, targeting glycolysis-associated pathways has emerged as a prominent area of investigation for radiosensitization (see Table 1) [135,136,137,138].

### 5.1. Hexokinase

Hexokinase (HK), the first enzyme in glycolysis, phosphorylates glucose to glucose-6-phosphate (G6P), initiating the glycolytic pathway and acting as a central regulatory hub in tumor metabolic reprogramming [155,156]. HK2, which is overexpressed in various tumors, plays a pivotal role in radiosensitization. By regulating glycolysis, HK2 meets the high-energy demands of tumors, supporting cell survival under radiation stress. Its overexpression accelerates glycolysis, increases ATP production, and maintains tumor viability during irradiation [134]. HK2 also interacts with mitochondrial VDACs to modulate redox homeostasis and limit ROS accumulation, increasing radiotolerance. Through these mechanisms, HK2 coordinates energy metabolism to sustain rapid proliferation and influence tumor radiosensitivity [141].

Bergenin (at 60 μM) suppresses HK2 expression (as confirmed by Western blotting) in radioresistant OSCC cells (CAL27IR and SCC25IR, established by serial exposure to 2 Gy/day of radiation up to a total dose of 80 Gy), thereby restoring radiosensitivity (as evidenced by colony formation assays), reducing proliferation and glycolysis, and promoting apoptosis [142]. The FDA-approved antifungal agent ketoconazole also inhibits HK2, thereby enhancing the efficacy of radiotherapy [143]. Overall, targeting HK2 significantly increases tumor radiosensitivity and shows promise for improving therapeutic outcomes.

### 5.2. PFK-1

PFK-1, a rate-limiting enzyme in glycolysis, catalyzes the conversion of F6P to fructose-1,6-bisphosphate, functioning as a pivotal regulatory checkpoint [157,158,159]. By controlling the glycolytic flux, PFK-1 fulfills the energy and biosynthetic requirements of tumor cells, driving metabolic reprogramming. Owing to its central role in tumor metabolism, PFK-1 is a promising target for radiosensitization strategies.

PFK-1 inhibitors reprogram tumor metabolism and improve the efficacy of radio- and chemotherapy. Among them, 3-(3-pyridyl)-1-(4-pyridyl)-2-propen-1-one (3PO) selectively inhibits PFKFB3, lowering fructose-2,6-bisphosphate (F2,6BP) levels and reducing glycolytic flux. 3PO preferentially targets Ras-transformed cells, suppressing adenocarcinoma growth. In combination with ascorbic acid, it synergistically induces ROS-dependent apoptosis [144]. Tryptolinamide (TLAM), another PFK-1 inhibitor, shows therapeutic promise in cells harboring mutant mitochondrial DNA by promoting mitochondrial respiration and restoring neural differentiation. TLAM inhibits PFK-1 to activate AMPK-mediated fatty acid oxidation, stimulate OXPHOS, and divert glycolytic intermediates into the pentose phosphate pathway (PPP), increasing antioxidant capacity [145]. Altered PFK-1 activity in tumors diverts glycolytic intermediates to the pentose phosphate pathway (PPP), supporting biosynthetic demands [160].

Genetic factors also influence PFK-1 activity. Regulators such as YY1, synoviolin, shRNA-507, SNAI, miR-520a/b/e, miR-128, and β-miR-6517 suppress tumorigenesis by modulating PFK-1 expression [161]. Furthermore, PFK-1 is regulated by metabolites, including ATP, ADP, AMP, and citrate, and by post-translational modifications. These multifaceted regulatory mechanisms support the development of novel PFK-1 inhibitors [162]. Targeting PFK-1 activity offers a strategic approach to reprogramming tumor metabolism and increasing radiosensitivity.

### 5.3. Pyruvate Kinase

Pyruvate kinase (PK), the final enzyme in glycolysis, catalyzes the conversion of PEP to pyruvate, generating ATP in the process [163,164]. In tumors, the M2 isoform (PKM2) predominates and plays a central role in metabolic reprogramming [165,166]. PKM2 exists in two conformations: an active tetramer and a less active dimer. The tetrameric form efficiently channels pyruvate into the TCA cycle, whereas the dimeric form favors lactate production, sustaining aerobic glycolysis [91]. The dimeric PKM2 can translocate to the nucleus, where it acts as a protein kinase to phosphorylate target proteins and promote tumor proliferation [167]. Owing to its dual metabolic and signaling functions, PKM2 is a promising target for radiosensitization.

Small-molecule inhibitors, such as shikonin, suppress tumor growth by inhibiting PKM2 and increasing ROS levels [146]. Activators (e.g., TEPP-46, DASA-58) stabilize PKM2 tetramers, elevating enzymatic activity to shift tumors from aerobic glycolysis to OXPHOS, improving radiotherapy efficacy [147,148,168]. PKM2 knockdown via siRNA/shRNA increases radiosensitivity in multiple tumor models, including glioma and lung cancer. shRNA-mediated PKM2 targeting combined with radiotherapy inhibits lung cancer proliferation and increases γ-H2AX expression [149]. PKM2-targeting shRNA increases leukemia cell radiosensitivity by suppressing NF-κB/HIF-1α signaling, decreasing glycolytic activity, and reducing stemness and DNA repair capacity [94]. Thus, modulation of PKM2 activity reprograms tumor metabolism and offers a viable strategy to increase radiosensitivity.

## 6. Clinical Applications of Glucose Metabolic Reprogramming

Glycolytic reprogramming is a hallmark of tumors and metabolic disorders, in which it promotes disease progression and influences therapeutic outcomes by regulating energy metabolism pathways, such as glycolysis and the pentose phosphate pathway, and key metabolic enzymes. The integration of radiotherapy and glycolytic modulation has become an essential focus in clinical research.

### 6.1. Glycolytic Pathway Intervention Strategies

Strategies targeting the glycolytic pathway encompass multiple therapeutic approaches. In targeted therapy, nano-delivery systems directed at YAP1/TXNIP, such as verteporfin-loaded PLGA nanoparticles, suppress synoviocyte glycolysis, reduce macrophage infiltration, and mitigate inflammatory microenvironments in diabetic osteoarthritis [150]. Among stem cell technologies, chemically induced pluripotent stem cells (CiPSCs) provide innovative options for treating metabolic diseases. Deng Hongkui’s group generated human CiPSCs in under 10 days and subsequently differentiated them into pancreatic β-cells for transplantation in type 1 diabetes. The transplant recipients achieved insulin independence by day 75, with efficacy sustained for over a year. This approach accelerates epigenetic reprogramming via the histone modifiers KAT3A/B and KAT6A, supporting personalized therapeutic applications [152]. Metabolic–immune synergy is also a viable approach: CRISPR activation (CRISPRa) promotes adipocyte browning, increasing the competitive use of glucose and fatty acids and suppressing breast and pancreatic tumor growth [153]. Such cross-regulatory interventions hold promise for integration with immune checkpoint inhibitors.

### 6.2. Radiotherapy–Glycolysis Co-Targeting

Co-targeting glycolysis and radiotherapy significantly improves therapeutic efficacy and helps overcome radioresistance. In laryngeal cancer, ionizing radiation induces GLUT-1 overexpression, contributing to radioresistance, whereas GLUT-1 antisense oligonucleotides increase radiosensitivity [169]. However, there are obstacles to clinical translation, including oligonucleotide delivery efficiency and potential off-target effects. Similarly, in HepG2 cells, radiotherapy increases lactate, alanine, and glucose levels, thereby promoting glycolysis and resistance to radiation. These findings require validation in diverse tumor models, given intertumoral metabolic heterogeneity. Metabolic intervention in these contexts significantly augments radiotherapy outcomes [154]. Radiotherapy–glycolysis co-targeting is a potent therapeutic approach that simultaneously modulates tumor energy metabolism and DNA repair capacity. However, potential toxicity to normal tissue resulting from metabolic disruption warrants careful investigation. Further research should focus on optimizing delivery strategies and validating safety profiles during clinical translation to improve patient prognosis.

## 7. Conclusions and Future Perspectives

Building on this foundation of glycolytic reprogramming, we focus on how the Warburg effect-driven metabolic shift specifically enables radioresistance through modulation of DNA repair, cell cycle dysregulation, and TME remodeling. Elucidating these mechanisms is essential for designing strategies to counteract radiotherapeutic resistance.

Radioresistance driven by glycolytic reprogramming operates through three principal mechanisms: (1) increased glycolysis supplies the energy and metabolic intermediates necessary for proliferation while modulating redox balance and signaling pathways to promote resistance; (2) upregulation of glycolytic enzymes and lactate production increases DNA DSB repair, mitigating radiation-induced damage; and (3) provision of energy and metabolites facilitates cell cycle progression, ultimately increasing radiotolerance.

Targeting glycolytic reprogramming shows significant therapeutic promise. The inhibition of key glycolytic enzymes, such as HK and PKM2, has been shown to elevate tumor radiosensitivity. HK inhibitors, including 3-bromopyruvate (3BP), suppress glycolysis and improve radiotherapeutic outcomes. Combining radiotherapy with metabolic interventions is highly efficacious, with glucose analogs such as 2-deoxy-D-glucose (2-DG) effectively inhibiting glycolysis to potentiate radiosensitivity. Furthermore, nanomaterial-based delivery systems, such as PLGA/PEG polymers carrying anti-glycolytic agents like LDHA- or GLUT1-targeting siRNAs, significantly amplify the effects of radiosensitization.

Future research should focus on developing innovative strategies that target glycolytic reprogramming and tumor heterogeneity concurrently. To this end, it is crucial to utilize technologies such as single-cell metabolomics and spatial transcriptomics to thoroughly analyze and delineate distinct metabolic subtypes within tumors, thereby laying a foundation for the design of interventions tailored to specific subpopulations. Promising research directions include the development of rational combination therapies, such as combining glycolytic inhibitors, like those targeting HK2 or LDHA, with immune checkpoint blockers to simultaneously disrupt the tumor’s energy supply and reverse immunosuppression, or co-targeting alternative pathways such as glutamine metabolism to counteract compensatory resistance. Concurrent advances in precision medicine based on metabolic profiling—using FDG-PET imaging and liquid biopsies for patient stratification—will help identify individuals most likely to benefit from glycolysis-targeted therapies. Furthermore, the development of multi-targeting nanocarriers capable of co-delivering radiosensitizers, metabolic inhibitors, and immunomodulators is a key technological approach to addressing intratumoral metabolic heterogeneity and enhancing drug delivery efficiency. Ultimately, validating the safety and efficacy of these integrated strategies through well-designed clinical trials is essential for successfully translating metabolic interventions into clinical practice.

In conclusion, glycolytic reprogramming contributes to radioresistance by promoting glycolysis, increasing lactate production, and increasing DNA repair capacity. Targeting glycolytic enzymes and integrating metabolic modulation with radiotherapy have strong potential for overcoming resistance. Advancing this field will depend on developing novel strategies, addressing tumor heterogeneity, and facilitating clinical application. This review highlights the critical role of glycolytic reprogramming in radiotherapy resistance and provides a framework for future research.

## Figures and Tables

**Table 1:** Summary of glycolysis-targeting therapeutic strategies and clinical applications.

Category	Target	Strategy Type	Specific Strategy/Compound	Clinical Stage	Reference
Enzyme-Targeted Strategies	GLUT1	Small molecule inhibitor	Oleanolic acid	Preclinical	[13]
		Genetic intervention	siRNA knockdown of GLUT1	Preclinical	[139]
	LDHA	Nano technology		Mostly experimental	[140]
		Genetic intervention	siRNA knockdown of LDHA	Mostly experimental	[38]
	HK2	Small molecule inhibitor	3BP *, Bergenin	Preclinical	[141,142]
		Small molecule inhibitor	Ketoconazole	Preclinical	[143]
	PFK-1	Small molecule inhibitor	3PO, Tryptolinamide (TLAM)	Preclinical	[144,145]
	PKM2	Small molecule inhibitor	Shikonin	Preclinical	[146]
		Small molecule activator	TEPP-46, DASA-58	Preclinical	[147,148]
		Genetic intervention	siRNA knockdown of PKM2	Mostly experimental	[94,149]
Platform and Combination Strategies	Multiple	Nanocarrier delivery	LDHA/GLUT1-targeting siRNA	Mostly experimental	[140,150]
		Broad-spectrum inhibition	2-DG	Clinical trials	[151]
		Stem cell technology	CiPSCs	Clinical trials	[152]
		Metabolic–immune synergy	CRISPR activation promotes browning of adipocytes	Mostly experimental	[153]
		Combination therapy	Radiotherapy and glycolysis inhibitors	Preclinical to clinical translation	[154]

* 3BP = 3-bromopyruvate; 3PO = 3-(3-pyridyl)-1-(4-pyridyl)-2-propen-1-one; CiPSCs = chemically induced pluripotent stem cells.

## Data Availability

No new data were created or analyzed in this study.
